# Perspective on Melatonin Use for Sleep Problems in Autism and Attention-Deficit Hyperactivity Disorder: A Systematic Review of Randomized Clinical Trials

**DOI:** 10.7759/cureus.8335

**Published:** 2020-05-28

**Authors:** Tarun Parvataneni, Sushma Srinivas, Kaushal Shah, Rikinkumar S Patel

**Affiliations:** 1 Psychiatry, Siddavanahalli Nijalingappa Medical College and HSK Hospital and Research Centre, Bagalkot, IND; 2 Psychiatry, A.J. Institute of Medical Sciences and Research Centre, Mangalore, IND; 3 Psychiatry, Griffin Memorial Hospital, Norman, USA

**Keywords:** melatonin, ramelteon, efficacy, safety study, rct, clinical trial, sleep problems, insomnia, child and adolescent psychiatry

## Abstract

Melatonin is a hormone produced by the pineal gland and is available over the counter for treating sleep problems in the pediatric population. We conducted a systematic review of randomized clinical trials (RCTs) on MEDLINE and included six studies that met our inclusion criteria. RCTs were conducted in patients from two to 18 years of age with a diagnostic and statistical manual of mental disorders (DSM)-IV diagnosis of autism spectrum disease (ASD) and/or attention-deficit hyperactivity disorder (ADHD) in both short-term and long-term RCTs ranging from eight-week to 52-week studies. The mean difference in the children’s sleep disorder showed statistically significant improvement in sleep duration and sleep latency onset compared to the placebo. Overall, a high response rate was observed in the melatonin group compared to the placebo in treating sleep problems in children. Melatonin is a well-tolerated and safe medication in the dose range of 2-10 mg/day in the child and adolescent population.

## Introduction and background

Sleep problems are increasingly becoming prevalent in children, as recent data found that about 50% of children from four to 12 years old have different sleep issues [1]. Sleep disturbances co-occur with psychiatric disorders including attention-deficit hyperactivity disorder (ADHD), autism spectrum disease (ASD), anxiety disorders, and major depressive disorder (MDD) [2]. As per a recent nationwide study, boys with ASD have two times higher likelihood of comorbid ADHD, with prevalence in white adolescents [3].

A higher proportion of patients with ASD have difficulty in sleep onset (53%), restless sleep (40%), nighttime awakening (34%), and difficulty in arousal from sleep (32%) [4]. The prevalence of sleep problems in ADHD patients is 73.3%, ranging from mild sleep disturbances (28.5%) to moderate and severe sleep disturbances (44.8%) such as frequent nighttime awakening, difficulty falling asleep, and circadian rhythm disturbance [5]. The prevalence of sleep problems in MDD is about 72.7% including insomnia, hypersomnia, or both, and children with anxiety disorders have nightmares, tiredness without exertion, and trouble sleeping [6-8].

Comorbid sleep problems have an impact on the daily functionality of children by an increase in externalizing and internalizing behaviors and an increase in autistic-like behaviors. Also, an increase in emotional and hyperactive symptoms can lead to impairment in academic and social functioning and maintaining relationships [7,9-10]. As these problems have a greater impact on a child’s growth and development, it is very important to diagnose and intervene at an earlier stage.

Based on past studies, non-pharmacologic treatment for sleep problems in children with psychiatric disorders includes behavioral therapy, like graduated extinction, bedtime routine, scheduled awakening, positive routine, and parent education [11-13]. Among pharmacological interventions, the most commonly used medications in the pediatric population are melatonin, trazodone, benzodiazepines, and antidepressants [11-13].

In our systematic review, we will do a qualitative synthesis of past randomized controlled trials (RCTs) conducted on melatonin in the pediatric population and to evaluate the overall efficacy and safety of melatonin for managing insomnia in ASD and neurodevelopmental disorder.

## Review

Study search strategy and selection

**Figure 1:**
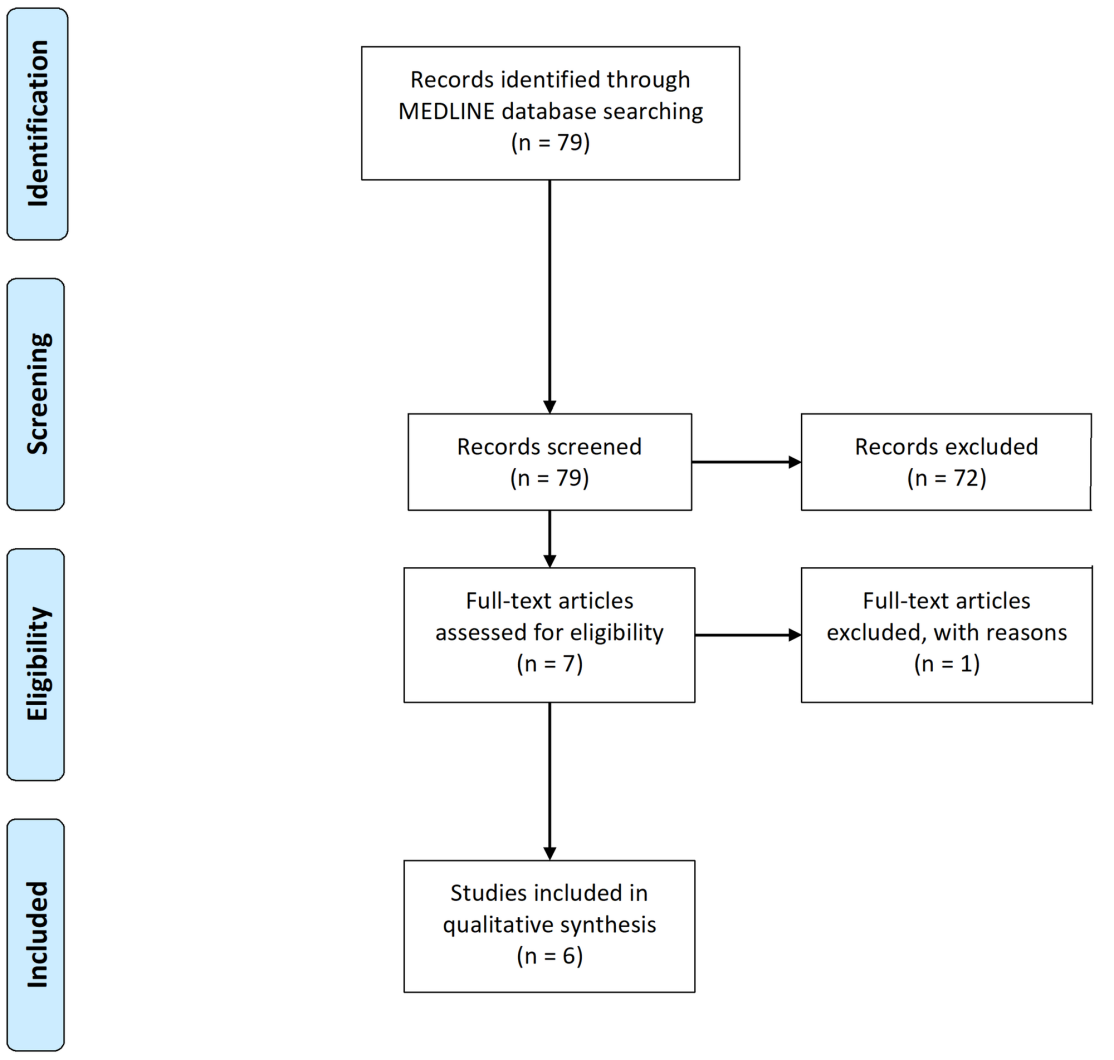
Systematic review of studies n, Number of studies

The MEDLINE database was used to identify clinical trials, clinical studies, multicenter studies, or observational studies published in English from April 19, 2010 to April 15, 2020 with MeSH terms "child" or "adolescent". The search strings were "melatonin" and "autism" or "autistic spectrum" or "developmental disorders" that yielded 79 studies. The screening was done independently by two authors (RSP, TP) using the Preferred Reporting Items for Systematic Reviews and Meta-analyses Statement (PRISMA) guidelines. The titles and abstracts were screened, based on the purpose of our study objective, and finally included seven studies. After reading the full text, a total of six studies met the criteria for our systematic review and were included as shown in Figure 1.

Mechanism of action

Melatonin or 5-methoxy N-acetyltryptamine is synthesized by the pineal gland from amino acid tryptophan by the first hydroxylation and then decarboxylation to form serotonin [14]. Melatonin production in the body is increased during darkness and reduced in light and so controls daily sleep and wakeful cycle [15]. Melatonin acts via MT1 receptors present in the suprachiasmatic nucleus (SCN) and controls the circadian rhythm [16].

Melatonin acts via MT1 G-protein coupled receptor that is pertussis toxin (PTX)-sensitive (Gi) and PTX insensitive (Gq). Activation of PTX-sensitive receptors inhibits cAMP, protein kinase A (PKA), and cAMP-responsive element-binding formation (CREB) [17]. SCN melatonin inhibits phosphorylation CREB, which leads to a change in circadian rhythm [18-19]. Similar to the MT1, MT2 receptor also inhibits the cAMP formation and cGMP formation. MT2 receptor interacts with diacylglycerol increases protein kinase C (PKC) in SCN, which affects the circadian clock [20].

Efficacy

Five studies conducted in an outpatient setting evaluated the efficacy of melatonin on sleep patterns in ASD [21-25]. Double-blinded-RCT (DB-RCT) conducted by Maras et al. included 95 pediatric patients with ASD and 51 patients received prolonged-release melatonin (2-10 mg/day) for 52 weeks and compared with placebo (N = 44). There was a statistically significant mean difference in the reduction of sleep latency (P<0.001) [21]. Similar results were also seen in a short-term study conducted by Gringras et al. [22]. An overview of the study design of included RCTs in shown in Table 1.

**Table 1:** Study design N, number of patients; ASD, autism spectrum disorder; ADHD, attention-deficit/hyperactivity disorder; OP, outpatient; CBT, Cognitive behavioral therapy; MTP, methylphenidate *Intervention group with CBT in Cortesi et al. and methylphenidate in Mohammadi et al. study. [20-25]

Study	Study details			Melatonin		Control	
	Demographic	Diagnosis	Trial	N	Dosage	N	Dosage
Malow et al.	Total N: 23 Age: 3-12 years	ASD	Length: 14 weeks, OP setting	23	1-6 mg	None	
Cortesi et al.	Total N:66 Age: 4-10 years	ASD	Length: 12 weeks, OP setting	34	3 mg*	32	Similar dosage of melatonin
Gringras et al.	Total N: 119 Age: 2-17 years	ASD	Length: 13 weeks, OP setting	58	2-5 mg	61	Similar dosage of placebo
Maras et al.	Total N: 95 Age: 2-17 years	ASD	Length: 52 weeks, OP setting	51	2-10 mg	44	Similar dosage of placebo
Schroder et al.	Total N: 119 Age: 2-17 years	ASD	Length: 13 weeks, OP setting	58	2-5mg	61	Similar dosage of placebo
Mohammadi et al.	Total N: 50 Age: 7-12 years	ASD and ADHD	Length: 8 weeks, OP setting	26	3-6 mg*	24	MTP only

Cortesi et al. included 66 children (age, 4-10 years) with ASD in their DB-RCT and found that patients on both cognitive-behavioral therapy (CBT) and melatonin improved significantly compared to those in the melatonin-only group in terms of reduction in sleep onset latency and wakefulness after sleep onset, and increase in total sleep duration [23]. Schroder et al. found that ASD patients managed with prolonged release of melatonin showed improvement in externalizing behavior. This was measured by the strength and difficulty questionnaire (SDQ) with a treatment difference of -0.74 but was statistically not significant (P = 0.076) [25]. This study did find that melatonin at a higher dose above 10 mg is also not efficacious in improving sleep quality [25].

DB-RCT conducted by Mohammadi et al. in children with ADHD and ASD (N = 50) showed that melatonin did not show statistically significant improvement in total duration of sleep compared to control groups that did not receive melatonin (P = 0.19) [26]. Only one study did not have a placebo group, as Malow et al. compared the pre- and post-treatment scores for children's sleep habit questionnaire [24]. They found a statistically significant improvement in sleep onset, total sleep duration, and reduced night walking, but no significant difference in sleep anxiety and parasomnias [24]. Outcomes of sleep quality between melatonin and control groups have been described in Table 2.

**Table 2:** Impact of melatonin on sleep quality Rx: treatment; SD: standard deviation; SDQ: strengths and difficulties questionnaire; WASO: wakeful after sleep onset. *This study used children's sleep habit questionnaire, and higher values indicate more difficulties. [20-25]

Study	Outcome	Melatonin		Control		P-value
		Pre-Rx score: mean (SD)	Post-Rx score: mean (SD)	Pre-Rx score: mean (SD)	Post-Rx score: mean (SD)	
Malow et al.*	Sleep onset delay	2.6(0.6)	1.3(0.6)	none		<0.0001
	Sleep duration	6.4(1.8)	3.7(1.3)			<0.0001
	Sleep total	55.2(6.9)	45.1(4.7)			<0.0001
	Sleep anxiety	6.8 (1.9)	6.3 (1.7)			0.270
	Night walking	5.3 (1.9)	4.3 (1.4)			0.023
	parasomnia	9.7 (2.0)	9.2 (2.1)			0.780
	Sleep-disordered breathing	3.8 (1.2)	3.5 (0.6)			0.170
	Daytime sleepiness	14.1 (2.4)	12.6 (2.7)			0.129
	Bedtime resistance	10.7 (4.2)	8.3 (2.3)			0.008
Cortesi et al.	Sleep total (minutes)	410.3 (45.1)	481.1 (33.2)	413.0 (45.1)	416.2 (43.6)	<0.001
	Sleep onset latency (minutes)	81.2 (32.4)	45.2 (23.2)	78.2 (33.8)	79.6 (31.9)	<0.001
	WASO (minutes)	73.7 (45.0)	42.2 (22.4)	69.75 (45.21)	70.2 (42.8)	<0.001
Gringras et al.	Total sleep time (minutes)	457.2	508.8 (10.5)	459.9	478.6 (10.8)	0.034
	Sleep latency (minutes)	95.2	57.3 (6.8)	98.8	86.2 (7.0)	0.011
Maras et al.	Sleep latency (minutes)	95.2	46.6 (10.2)	98.8	65.2 (8.3)	<0.001
Schroder et al.	SDQ	Mean difference -0.57 (0.3)		Mean difference 0.16 (0.3)		0.076
Mohammadi et al.	Total sleep duration (in hours)	8	8.5	8.8	8.3	0.19

Adverse events

Melatonin is safe and widely used for sleep disorders. As per a 52-week long-term study, the most common side effects with melatonin include fatigue (18.9%), vomiting (16.8%), mood swings (13.7%), and upper respiratory infection (10.5%) [21].

Limitations of studies

The studies mentioned in our systematic review had some limitations. RCT by Malow et al. did not compare the results of the melatonin group with placebo [24]. DB-RCT by Cortesi et al. was conducted for 12 weeks that only validates short-term efficacy of melatonin, but on the contrary, Maras et al. conducted DB-RCT for the 52-week period to justify melatonin’s long-term efficacy and safety [21,23]. We cannot generalize the study results in ASD with/without ADHD patients to children with other psychiatric illnesses. Also, participants were compliant with medication after receiving parental consent, which may not be the scenario in the general population. In DB-RCT by Schroder et al., no supporting evidence existed for change in internalizing behavior evaluated by caregivers; the small sample size was not efficiently powered to detect the difference in sleep effect and behavior [25]. In the DB-RCT by Gringras et al. sleep monitoring was measured by actigraphy due to refusal to wear the device by the patient [22]. RCT by Maras et al. was open-label and a follow-up study of Gringras et al. study, and so it is possible that the improvement in the intervention group could be due to the efficacy of melatonin versus spontaneous remission in this long-term study [21-22].

## Conclusions

Patients with ASD and/or ADHD showed improvement in total sleep time, sleep latency and improved uninterrupted sleep with melatonin, and augmented by combination management with melatonin and CBT. There was an improvement in externalizing behavior and subsequent improvement in the caregiver’s quality of life. Melatonin is a well-tolerated and safe medication in the dose range of 2-10 mg/day in the child and adolescent population. The efficacy and safety were proved by both short-term and long-term DB-RCTs.
